# Adenosine Receptor-Mediated Developmental Loss of Spike Timing-Dependent Depression in the Hippocampus

**DOI:** 10.1093/cercor/bhy194

**Published:** 2018-08-31

**Authors:** Mikel Pérez-Rodríguez, Luis E Arroyo-García, José Prius-Mengual, Yuniesky Andrade-Talavera, José A Armengol, Eva M Pérez-Villegas, Paloma Duque-Feria, Gonzalo Flores, Antonio Rodríguez-Moreno

**Affiliations:** 1 Laboratory of Cellular Neuroscience and Plasticity, Department of Physiology, Anatomy and Cell Biology, Universidad Pablo de Olavide, Seville, Spain; 2 Instituto de Fisiología, Benemérita Universidad Autónoma de Puebla, 72570 Puebla, Mexico; 3 Human Anatomy and Embryology Unit, Department of Physiology, Anatomy and Cell Biology, Universidad Pablo de Olavide, Seville, Spain

**Keywords:** adenosine receptors, astrocytes, hippocampus, plasticity windows, spike timing-dependent plasticity

## Abstract

Critical periods of synaptic plasticity facilitate the reordering and refining of neural connections during development, allowing the definitive synaptic circuits responsible for correct adult physiology to be established. Presynaptic spike timing-dependent long-term depression (t-LTD) exists in the hippocampus, which depends on the activation of NMDARs and that probably fulfills a role in synaptic refinement. This t-LTD is present until the third postnatal week in mice, disappearing in the fourth week of postnatal development. We were interested in the mechanisms underlying this maturation related loss of t-LTD and we found that at CA3–CA1 synapses, presynaptic NMDA receptors (pre-NMDARs) are tonically active between P13 and P21, mediating an increase in glutamate release during this critical period of plasticity. Conversely, at the end of this critical period (P22–P30) and coinciding with the loss of t-LTD, these pre-NMDARs are no longer tonically active. Using immunogold electron microscopy, we demonstrated the existence of pre-NMDARs at Schaffer collateral synaptic boutons, where a decrease in the number of pre-NMDARs during development coincides with the loss of both tonic pre-NMDAR activation and t-LTD. Interestingly, this t-LTD can be completely recovered by antagonizing adenosine type 1 receptors (A_1_R), which also recovers the tonic activation of pre-NMDARs at P22–P30. By contrast, the induction of t-LTD was prevented at P13–P21 by an agonist of A_1_R, as was tonic pre-NMDAR activation. Furthermore, we found that the adenosine that mediated the loss of t-LTD during the fourth week of development is supplied by astrocytes. These results provide direct evidence for the mechanism that closes the window of plasticity associated with t-LTD, revealing novel events probably involved in synaptic remodeling during development.

## Introduction

One of the most interesting properties of the mammalian brain is its ability to change in response to experience. This phenomenon was called plasticity more than a century ago (Cajal 1894) and it is involved in the organization of cortical maps during development, as well as in learning and memory processes in adults (Malenka and Bear 2004; Citri and Malenka 2008; Takeuchi et al. 2014). The forms of plasticity most extensively studied are the long-term potentiation (LTP) and long-term depression (LTD) of synaptic transmission. Permissive and critical periods of plasticity exist during development (stages of maturation during the lifespan of organisms), windows in which intense activity (sensory)-dependent plasticity occurs and environmental experiences have the greatest impact on brain circuitry. In these windows of plasticity, the reordering and refinement of neural connections drives the formation of the definitive circuits responsible for correct adult physiology (Hensch 2005). Moreover, the closing of these windows is associated with the loss of plasticity at particular synapses (Hensch 2004, 2005). Spike timing-dependent plasticity (STDP) is a Hebbian form of long-term synaptic plasticity detected in all species studied from insects to humans, and it is a strong candidate to underlie circuit remodeling during development, as well as for learning and memory (see Feldman 2012 for a review). In STDP, the order and precise millisecond timing of presynaptic and postsynaptic action potentials (spikes) determines the direction and magnitude of the synaptic change. Thus, timing-dependent LTP (t-LTP) occurs when a presynaptic spike is followed by a postsynaptic spike within 10–15 ms, whereas timing-dependent LTD (t-LTD) is induced when this order is reversed (Feldman 2012).

A presynaptic form of spike timing-dependent LTD (t-LTD) that requires the activation of presynaptic NMDA receptors (pre-NMDARs) has been described in the hippocampus, and in the visual and somatosensory cortices (Corlew et al. 2007; Banerjee et al. 2009; Andrade-Talavera et al. 2016; Bouvier et al. 2018). In the hippocampus, t-LTD is dependent on postsynaptic Ca^2+^, L-type voltage-dependent Ca^2+^ channels, mGlu5 receptor activation, phospholipase C and postsynaptic IP_3_ receptor-mediated Ca^2+^ release from internal stores, postsynaptic endocannabinoid (eCB) synthesis, activation of CB1 receptors and astroglial signaling, delivering the d-serine coagonist to NMDARs during the induction of t-LTD. This t-LTD is expressed presynaptically, as indicated by the analysis of trial-to-trial fluctuation in excitatory postsynaptic potentials (EPSPs) (Andrade-Talavera et al. 2016).

These presynaptic forms of t-LTD disappear in the first weeks of development (Corlew et al. 2007; Banerjee et al. 2009; Andrade-Talavera et al. 2016), by the fourth week of postnatal development in the mouse hippocampus, although how this loss is brought about is not known. Moreover, it is unclear whether the closing of this window of plasticity in the fourth week of development is reversible or not. Determining the mechanisms that produce the closure of plasticity windows is important when studying the brain responses to experience and injury. Indeed, defining such processes may have important implications for brain repair, sensorial recovery, the treatment of neurodevelopmental disorders and even, for educational policy.

To better understand the mechanisms driving the loss of plasticity during development, we have studied the mechanisms involved in the loss of t-LTD observed with maturation at Schaffer collateral (SC)—CA1 synapses in the mouse hippocampus using whole-cell patch-clamp recordings and immunogold electron microscopy (EM). We found that presynaptic t-LTD can be induced at hippocampal CA3–CA1 synapses in young mice (P8–P21), although this presynaptic form of plasticity is lost in the fourth week of development (P22–P30), and we confirmed that this form of plasticity does not require postsynaptic NMDARs. Furthermore, we found that pre-NMDARs are tonically active at P13–P21 but not at the end of this critical period (P22–P28), coinciding with the loss of t-LTD. We found a decrease in the number of pre-NMDARs during development that coincides with the loss of both tonic pre-NMDAR activation and t-LTD. In addition, we discovered that the developmental loss of t-LTD is reversed by antagonizing adenosine type 1 receptors (A_1_Rs), which also recovers the tonic pre-NMDAR activation lost at P22–P30. Conversely, the induction of t-LTD and tonic pre-NMDAR activation are impaired between P13 and P21 in the presence of an A_1_R agonist. Hence, enhanced inhibition mediated by the activation of A_1_Rs during development appears to be responsible for the loss of tonic pre-NMDAR activation and t-LTD. Finally, we found that the adenosine that mediates the loss of t-LTD in the fourth week of development is released by astrocytes in a calcium-dependent manner.

## Materials and Methods

### Ethical Approval

All animal procedures were carried out in accordance with the European Union Directive 2010/63/EU regarding the protection of animals used for scientific purposes and they were approved by the local Ethical Committees. C57BL/6 mice were obtained from Harlan Laboratories (Spain) and male mice aged postnatal day (P) 8–30 were used.

### Slice Preparation

Hippocampal slices were prepared as described previously (Rodríguez-Moreno et al. 1998; Andrade-Talavera et al. 2012; 2016). Briefly, mice were anesthetized with isoflurane (2%) and decapitated for slice preparation. The whole brain containing the 2 hippocampi was removed and placed in an ice-cold solution containing (in mM): NaCl, 126; KCl, 3; NaH_2_PO_4_, 1.25; MgSO_4_, 2; CaCl_2_, 2; NaHCO_3_, 26; and glucose, 10 (pH 7.2, 300 mOsm L^−1^). Transverse hippocampal slices (350 μm thick) were obtained on a vibrating blade microtome (Leica VT1000S) and they were maintained oxygenated (95% O_2_/5% CO_2_) in this solution for at least 1 h before use. All experiments were carried out at room temperature (22–25 °C) and during the experiments, the slices were continuously superfused with the solution indicated above.

### Electrophysiological Recordings

Whole-cell patch-clamp recording of pyramidal cells located in the CA1 field of the hippocampus were obtained under visual guidance by infrared differential interference contrast microscopy. The neurons were verified as pyramidal cells through their characteristic voltage response to a current step protocol. The neurons were recorded in current-clamp configuration with a patch clamp amplifier (Multiclamp 700B) and the data were acquired using pCLAMP 10.2 software (Molecular Devices). Patch electrodes were pulled from borosilicate glass tubes and they had a resistance of 4–7 MΩ when filled with (in mM): potassium gluconate, 110; HEPES, 40; NaCl, 4; ATP-Mg, 4; and GTP, 0.3 (pH 7.2–7.3, 290 mOsm L^−1^). Only cells with a stable resting membrane potential negative to −60 mV were assessed and the cell recordings were excluded from the analysis if the series resistance changed by more than 15% during the recording. During the experiments, the changes in Vm (1–3 mV) were corrected by imposing continuous current (10–30 pA) to maintain the membrane potential constant. CA3–CA1 connections were not cut and the few slices that showed epileptic activity were discarded. All recordings were low-pass filtered at 3 kHz and acquired at 10 kHz. In plasticity experiments, EPSPs were evoked alternately through 2 input pathways, the test and control, each at 0.2 Hz. The EPSPs were induced by 2 monopolar stimulation electrodes placed in the “stratum radiatum” using brief current pulses (200 μs, 0.1–0.2 mA). Stimulation was adjusted to obtain an EPSP peak amplitude of approximately 4–5 mV in control conditions. Pathway independence was assured by the lack of cross-facilitation when the pathways were stimulated alternately with a 50 ms interval. Plasticity was assessed through the changes in the slope of the EPSP, measured in its rising phase as a linear fit between time points corresponding to 25–30% and 70–75% of the peak amplitude under control conditions. Miniature responses were recorded in the presence of 500 nM TTX (Tetrotodoxin). The pipette solution in voltage-clamp experiments contained (in mM): CsCl 140; EGTA 0.2; HEPES 10.0; ATP-Mg 2.0; GTP-NaCl 0.3; QX-314 5.0, adjusted to pH 7.2 with NaOH. Voltage clamp experiments were performed in the presence of bicuculline (10 μM) and NBQX (10 μM), and NMDAR-mediated currents were recorded at +40 mV. Astrocytes were identified by their small soma size (about 12 μm), low resting potential (−81 ± 5 mV) and absence of action potentials.

### Plasticity Protocols

After establishing a stable EPSP baseline over 10 min, the test input was paired 100 times with a single postsynaptic spike. The single postsynaptic spike was evoked by a brief somatic current pulse (5 ms, 80–120 pA) and the control pathway was unstimulated during the pairing period. To induce t-LTD, the postsynaptic action potential was evoked within 18 ms before the onset of the EPSP. EPSP slopes were monitored for at least 30 min after the pairing protocol, and the presynaptic stimulation frequency remained constant throughout the experiment.

### Pharmacology

Pharmacological agents were purchased from:

Sigma Aldrich—BAPTA, bicuculline methobromide, TTX, paraformaldehyde, tannic acid, tergitol, d-serine and all the salts used to prepare the internal and external solutions; Tocris Bioscience—(+)-MK-801 maleate, D-AP5, 8-CPT, and CPA. Bio-Rad—glutaraldehyde.

### Data Analysis

The data were analyzed using the Clampfit 10.2 software (Molecular Devices) and the last 5 min of recording was used to estimate the changes in synaptic efficacy relative to the baseline. For paired-pulse ratio (PPR) experiments, 2 EPSPs were evoked for 30 s at the basal frequency at the beginning of the baseline recording, 40 ms apart, and again 30 min after the end of the pairing protocol. The PPR was expressed as the slope of the second EPSP relative to the slope of the first EPSP.

### Immunogold Electron Microscopy

Two P15 and 2 P30 mice were used for these studies. The mice were anesthetized deeply with pentobarbital (80 mg/kg, i.p.), and perfused transcardially for 10 min with ice cooled 2% paraformaldehyde and 1% glutaraldehyde in 0.1 M phosphate buffer (PB, pH 7.4). After dissection, coronal vibratome brain sections (50 μm) were obtained in PB at 4 °C, they were fixed in 2% paraformaldehyde and 0.1% glutaraldehyde in PB on ice, and 15 min after obtaining the sections, those containing the hippocampus were processed according to the osmium-free method of Phend et al. (1995). Briefly, after rinsing in 0.1 M maleate buffer (MB, pH 6), the sections were incubated for 30 min in 1% tannic acid in MB and block stained for 40 min on ice with 1% uranyl acetate in MB. After rinsing in cold MB, the sections were dehydrated with cold ethanol and acetone at room temperature, and flat-embedded in Durcupan (Fluka^®^). The hippocampus was trimmed from the flat-embedded sections and set in a resin block with cyanoacrylate. Sections (80–100 nm thick) were obtained on a Leica UC6 ultramicrotome and collected on 300 mesh hexagonal nickel grids treated with Coat-Quick. The grids were washed in 0.05 M Tris buffer with 0.9% NaCl and 0.005% Tergitol (TBST, pH 7.6), and then they were incubated overnight at room temperature with a rabbit monoclonal antibody against the NMDAR (GluN1R, 1:250, Millipore, AB9864R) diluted 1:100 in TBST (pH 7.6). After rinsing in TBST (pH 7.6), the sections were incubated for 1 h with a goat antibody against rabbit IgG F(ab′)2 conjugated to 10 nm gold particles (Aurion^®^, Electron Microscope Science, no. 25365) diluted 1:25 in TBST (pH 8.2). To avoid possible artifacts or precipitates, ultrathin sections were observed without uranyl acetate and lead citrate counterstaining on a Zeiss Libra 120 transmission electron microscope. The criteria used to analyze the presence of presynaptic and postsynaptic NMDARs (NR1) were those followed by Larsen et al. 2011. Briefly, in a 352 μm^2^ area of the stratum radiatum of each animal, we only considered asymmetric synapses with clear vesicles when assessing postsynaptic or presynaptic labeling. The criteria for positive labeling was the presence of gold particle/s within the synaptic density (pre-, post-, or both) or at a distance of less than 20 nm.

### Statistical Analysis

A normality and equal variance test was performed before applying statistical comparisons, which were performed with a paired or unpaired Student's *t*-test as appropriate. The data are expressed as the mean ± standard error of mean (S.E.M.) and *P* values less than 0.05 were considered significant.

## Results

### Pairing Presynaptic Activity With Single Postsynaptic Action Potentials at Low Frequency can Induce t-LTD in the Mouse Hippocampus at P13–P18, Which Requires Nonpostsynaptic NMDARs

**Figure 1.**
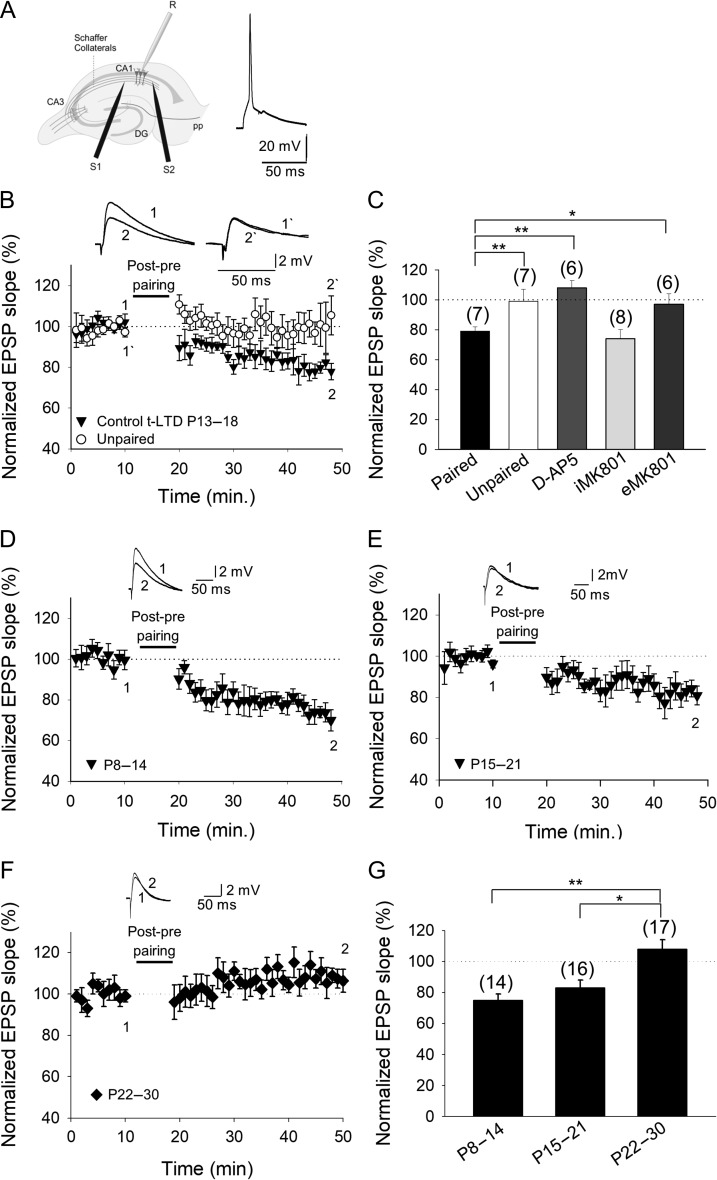
Input-specific spike timing-dependent plasticity in the CA1 region of the hippocampus is present at P8–P14 and P15–P21 but not at P22–P30. (*A*) Left, scheme showing the general experimental set-up: R, recording electrode; S1 and S2, stimulating electrodes; right, pairing protocol utilized (Δ*t*, time between EPSP onset and peak of spike). (*B*) Post–pre single-spike pairing protocol induced t-LTD. The EPSP slopes monitored in the paired (black circles) and unpaired pathway (open circles) are shown. Traces show the EPSP before (1) and 30 min after (2) pairing. Depression was only observed in the paired pathway. (*C*) Summary of the results. Note that t-LTD does not require postsynaptic NMDARs. In the presence of d-AP5 (50 μM), t-LTD was completely blocked with MK-801 added to the bath (eMK-801), yet t-LTD was not affected when MK-801 was loaded into the postsynaptic cell (iMK-801). The t-LTD is evident during the second (*D*) and third (*E*) week of development but it disappears during the fourth week (*F*). (*G*) Summary of the results, where the error bars represent the S.E.M. and the number of slices is shown in parentheses: *P < 0.05; **P < 0.01, unpaired Student’s *t*-test.

We first confirmed here that pairing presynaptic stimulation with single postsynaptic spikes at low frequency is sufficient to induce t-LTD at CA3–CA1 synapses. In slices prepared from the mouse hippocampus at postnatal (P) days 13–18, the EPSPs evoked by extracellular stimulation of SCs in the *stratum radiatum* were monitored by whole-cell recording of CA1 pyramidal cells as described previously (Fig. 1*A*) (Andrade-Talavera et al. 2016). Accordingly, t-LTD was induced in current-clamp mode using 100 pairings of single EPSPs and single postsynaptic spikes at 0.2 Hz. A post-before-pre pairing protocol (post–pre protocol), with a postsynaptic spike occurring ~18 ms before the presynaptic stimulation, induced robust t-LTD (79 ± 3%, *n* = 7), whilst an unpaired control pathway remained unchanged (99 ± 8%, *n* = 7: Fig. 1*B*,*C*).

Treating slices with the NMDAR antagonist d-2-amino-5-phosphonopentanoic acid (d-AP5) blocked the t-LTD produced by the post–pre protocol (108 ± 6%, *n* = 8 versus interleaved controls, 74 ± 8%, *n* = 5: Fig. 1*C*), indicating that t-LTD at P13–P18 requires NMDARs. To confirm that the NMDARs required for t-LTD are not postsynaptic, we repeated the pairing experiments after loading the postsynaptic neuron with the NMDAR blocker MK-801 via the recording patch pipette. As reported previously for these synapses (Andrade-Talavera et al. 2016), and as seen at neocortical synapses (Sjöström et al. 2003; Bender et al. 2006; Nevian and Sakmann 2006; Rodríguez-Moreno and Paulsen 2008; Rodríguez-Moreno et al. 2011, 2013, Banerjee et al. 2014) blocking postsynaptic NMDARs did not prevent t-LTD (76 ± 7%, *n* = 9; vs. interleaved controls, 72 ± 5%, *n* = 9: Fig. 1*C*), whereas adding MK-801 to the bath solution (500 μM or 1 mM) fully impaired the induction of t-LTD (Fig. 1*C*). Adding MK-801 either extracellularly or by loading the postsynaptic neuron completely blocked NMDAR-mediated currents recorded from the postsynaptic cell (Supplementary Fig. S1).

### Loss of t-LTD During the Fourth Week of Development

We studied the age profile of this form of t-LTD, confirming that as reported previously (Andrade-Talavera et al. 2016), t-LTD can be induced until P21 (72 ± 6%, *n* = 14 at P8–P14; 83 ± 5%, *n* = 16 at P15–P21: Fig. 1*D*,*E*,*G*), disappearing thereafter in the fourth week of development (at P22–P30, 106 ± 7%, *n* = 17: Fig. 1*F*,*G*). Hence, this form of t-LTD is clearly related to a specific developmental period as it is not induced after that.

### Presynaptic NMDARs are Tonically Active and They Facilitate Glutamate Release in the Hippocampus of Young (P13–P21) but not Older Mice (P22–P30)

Early in development, synapses frequently show a high probability of neurotransmitter release that switches to a low probability of neurotransmitter release as they mature, as seen in the somatosensory (Frick et al. 2007), auditory (Oswald and Reyes 2008), visual (Cheetham and Fox 2010), and prefrontal (González-Burgos et al. 2008) cortices. Pre-NMDARs are thought to be tonically active at some synapses during development in the entorhinal (Berretta and Jones 1996), visual (Larsen et al. 2011), and somatosensory (Brasier and Feldman 2008) cortices, and as seen indirectly in the hippocampus (Mameli et al. 2005). Thus, to determine the mechanisms involved in the closure of this early window of plasticity, we checked for tonic activation of pre-NMDARs at CA3–CA1 synapses in our experiments.

#### Presynaptic NMDARs Regulate Evoked Release at CA3–CA1 Synapses at P13–P21 but not at P22–P30

**Figure 2.**
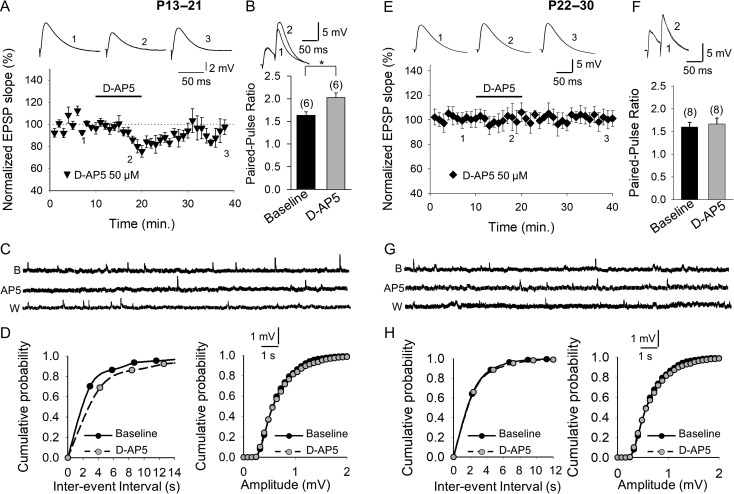
Presynaptic NMDARs are tonically active at CA3–CA1 synapses at P13–P21 but not at P22–P30. (*A*) With the postsynaptic neuron loaded with MK-801, the addition of D-AP5 decreases the slope of evoked EPSPs, an effect that was reversed after D-AP5 washout. The inset shows the EPSP traces at baseline (1), in the presence of D-AP5 (2) and after D-AP5 wash out (3). (*B*) The paired-pulse ratio increased in the presence of D-AP5. (*C*) Miniature EPSPs monitored during the baseline and after exposing neurons from slices of P13–P21 animals to D-AP5 in the presence of TTX (500 nM), and with the postsynaptic neuron loaded with MK-801 (1 mM). (*D*) Cumulative probability histograms showing that at P13–P21, D-AP5 reversibly decreases the mEPSP frequency but it does not affect the mEPSP amplitude. (*E*) D-AP5 does not affect the evoked EPSP slope at P22–P30. The inset shows the traces at baseline (1), in the presence of D-AP5 (2) and after D-AP5 wash out (3). (*F*) At P22–P30, the paired-pulse ratio is not affected by D-AP5. (*G*) Miniature EPSPs monitored at baseline, and during and after D-AP5 treatment of neurons from P22 to P30 mice in the presence of TTX, and with the postsynaptic neuron loaded with MK-801. (*H*) Cumulative probability histograms showing that D-AP5 does not affect mEPSP frequency or amplitude at P22–P30. The error bars indicate the S.E.M. and the number of slices is shown in parentheses: *P < 0.05, unpaired Student’s *t*-test.

While stimulating SC afferents, we studied the evoked EPSPs in whole-cell recordings obtained from CA1 neurons in transverse hippocampal slices from P13 to P21 C57BL/6 mice. To confirm that the NMDARs modulating these synapses were not postsynaptic, we loaded the postsynaptic neuron with MK-801 (1 mM). In these conditions, bath application of D-AP5 (50 μM) caused the EPSP slope to decrease (to 80 ± 3%, *n* = 6) and this modification was reversed on the washout of D-AP5 (Fig. 2*A*), indicating the existence of tonically active pre-NMDARs. In accordance with the presynaptic decrease in the probability of glutamate release caused by D-AP5, the PPR increased in the presence of D-AP5 (1.60 ± 0.08 baseline vs. 2.0 ± 0.1, *n* = 6 in D-AP5, *n* = 6, Fig. 2*B*). By contrast, D-AP5 did not affect the EPSP slope (101 ± 7%, *n* = 8, Fig. 2*E*) or PPR (1.6 ± 0.1 baseline, 1.66 ± 0.13 D-AP5, Fig. 2*F*) in hippocampal slices from P22 to P30 mice, indicating that pre-NMDARs are not tonically active at P22–P30 and that tonic activation is lost as development proceeds.

The presence (at P13–P21) or absence (at P22–P30) of tonic pre-NMDAR activation is not due to the changes in glutamate transporters with maturation, as there were no differences in the parameters recorded in the presence of the glutamate transporter blocker TBOA (50 μM) at P13–P21 and P22–P30. As such, robust t-LTD was observed at P13–P21 in the presence of TBOA (70 ± 6%, *n* = 6) and at P22–P30, no t-LTD was observed in slices treated with TBOA (110 ± 15%, *n* = 6, Supplementary Fig. S2A, B). Accordingly, t-LTD seems to be directly dependent on tonic activation of pre-NMDARs by glutamate as changing the probability of glutamate release by modifying the extracellular Ca^2+^ concentration directly affected t-LTD. In fact, t-LTD could not be induced at P13–P22 when the extracellular Ca^2+^ concentration was decreased to 1 mM (107 ± 8%, *n* = 6), whereas t-LTD at P22–P30 (when is normally lost) was rescued (79 ± 6%, *n* = 6) when the extracellular calcium concentration was increased to 4 mM (Supplementary Fig. S2B, C).

#### Pre-NMDARs Regulate mEPSP Frequency at P13–P21 but not at P22–P30

To study the effect of pre-NMDARs on spontaneous glutamate release at SCs, we again blocked postsynaptic NMDARs at P13–P21 by introducing MK-801 (1 mM) into the postsynaptic neuron. When we then measured the effects of D-AP5 (10 min.) on mEPSP frequency and amplitude (or slope) in the presence of TTX (500 nM), we found that D-AP5 produced a decrease in mEPSP frequency (baseline 0.37 ± 0.05 Hz, *n* = 6; D-AP5 0.22 ± 0.05 Hz, *n* = 6; interevent-interval, baseline 2.9 ± 0.1 s, *n* = 6; D-AP5 4.2 ± 0.2 s, *n* = 6: Fig. 2*C*,*D* and Supplementary Fig. S3), with no effect on mEPSP amplitude (Fig. 2*C*,*D* and Supplementary Fig. S3). This effect was specific to NMDARs as the effect was reversible, and no changes in frequency, amplitude or slope were found when the experiment was performed in the absence of D-AP5 (data not shown). Conversely, D-AP5 did not affect the mEPSP frequency in slices from P22–P30 mice (0.4 ± 0.04 baseline, 0.43 ± 0.05 D-AP5), nor the mEPSP amplitude (0.35 ± 0.01 mV baseline, 0.37 ± 0.02 D-AP5, *n* = 7: Fig. 2*G*,*H* and Supplementary Fig. S3). These results indicate that, as for evoked release, NMDARs are not tonically active at this later developmental stage, and that during development the tonic activation of pre-NMDARs is lost.

These data demonstrate a developmental loss in the ability of pre-NMDARs to facilitate glutamate release in the CA3–CA1 region of the hippocampus and a decrease in the glutamate release probability that parallels the loss of t-LTD.

### The Developmental Loss of Pre-NMDAR Activity Coincides With a Reduction in the Number of Pre-NMDARs

**Figure 3.**
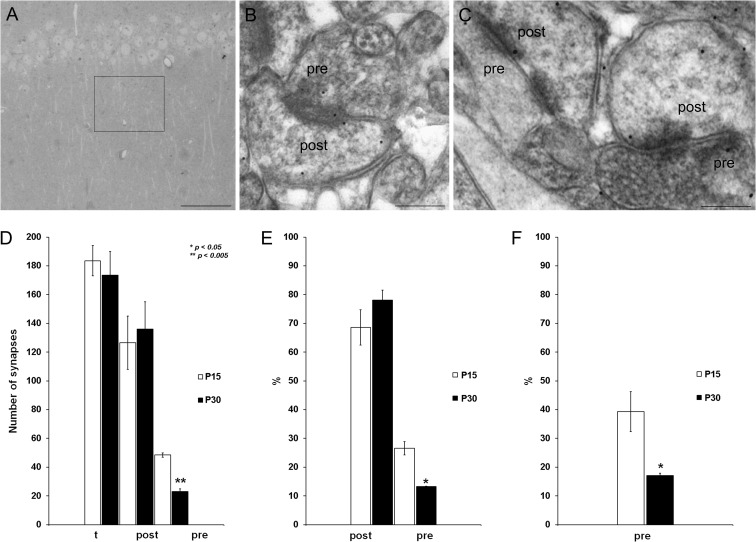
Presynaptic NR1 is downregulated during development. (*A*) Transmitted light photomicrograph illustrating the CA1 area of the mouse hippocampus analyzed (square). (*B*, *C*) Electron photomicrographs of the CA1 demonstrating the presence of NR1 immunolabeling (arrows) at both presynaptic (pre) and postsynaptic (post) sites at P15 (*B*) and P30 (*C*). Scale bars: 200 μm (*A*) and 200 nm (*B*, *C*). (*D*) The number of NR1 immunolabeled postsynaptic sites did not change from P15 to P30, whereas the number of immunolabeled presynaptic terminals decreased significantly at P30 compared with P15. (*E*) The proportion of presynaptic labeled terminals relative to the total number of synapses decreases with age, while the percentage of labeled postsynaptic terminals does not change significantly. (*F*) A statistically significant decrease was also observed when the proportion of presynaptic labeled terminals was compared with the number of NR1 labeled postsynaptic terminals: *t*, total number of synapses counted, *n* = 4 mice (2 for each age analyzed). The error bars represent the S.E.M., and >150 synapses per mouse and region were analyzed: *P < 0.05; **P < 0.01, unpaired Student’s *t*-test.

One possible explanation for the developmental loss of pre-NMDAR function is a reduction in the number of synapses with pre-NMDARs or in the number of pre-NMDARs at the existing synapses, as suggested in the visual cortex where a reduction in the number of pre-NMDARs does occur during development (Corlew et al. 2007; Larsen et al. 2011). To determine whether a similar decrease in the number of pre-NMDARs occurs in the hippocampus, we used immunogold EM to examine the presynaptic and postsynaptic NMDARs in the SC, assessing the distribution of the NR1 subunit (essential to NMDARs). A total of 356 synapses were analyzed in tissue from 2 P15 and P30 mouse brains (2 brains at P15, 183 synapses; and 2 brains at P30, 173 synapses, Fig. 3*A*), and NR1 immunolabeling was evident at both presynaptic (pre) and postsynaptic (post) sites in electron photomicrographs of both the P15 (Fig. 3*B*) and P30 (Fig. 3*C*) CA1. The number of NR1 immunolabeled postsynaptic sites did not vary from P15 (126 ± 18) to P30 (136 ± 19: Fig. 3*D*), whereas the number of immunolabeled presynaptic terminals decreased significantly (P15, 48 ± 1; P30, 23 ± 2: Fig. 3*D*). Similarly, the percentage of presynaptic labeled terminals decreased with age relative to the total number of synapses (P15, 26 ± 2%; P30, 13.0 ± 0.1%, Fig. 3*E*), while the number of those postsynaptic terminals did not change significantly (P15, 68 ± 6%; P30, 78 ± 3%: Fig. 3*E*). This significant decrease was also observed when the percentage of presynaptic labeled terminals was compared with the number of NR1 postsynaptic labeled terminals (P15, 39 ± 7%; P30, 17.0 ± 0.9%: Fig. 3*F*). These data confirm the anatomical existence of pre-NMDARs at SC synaptic boutons and also, a reduction in the number of pre-NMDARs during development that coincides with the loss of both tonic pre-NMDAR activation and t-LTD.

### The Loss of t-LTD is not Due to a Shift in the Coincidence Time Window Needed to Induce t-LTD

**Figure 4.**
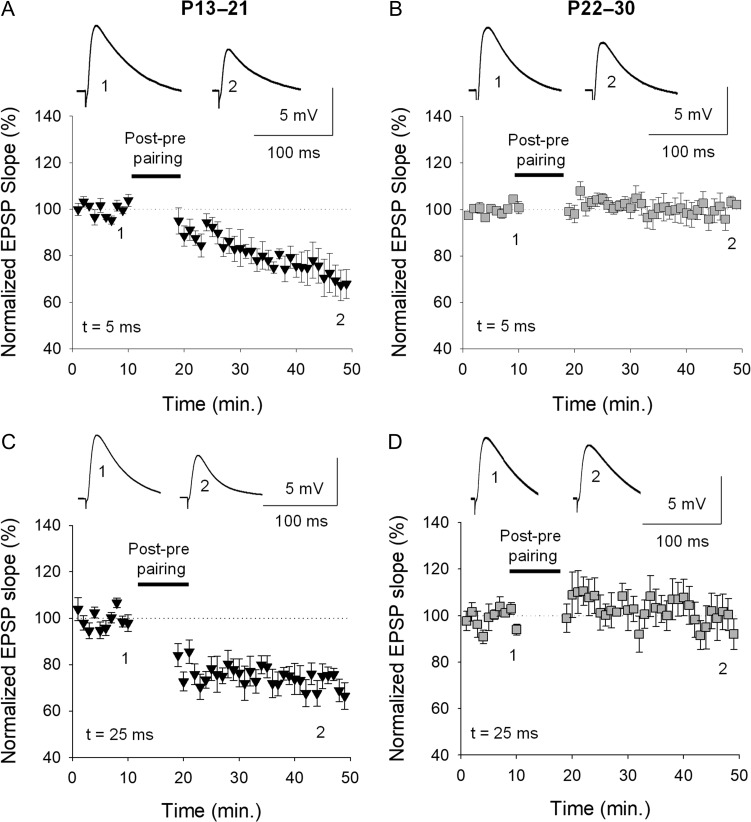
The loss of t-LTD is not due to a shift in the coincidence time window to induce t-LTD. Using timings between presynaptic and postsynaptic activity to induce t-LTD that were shorter and longer than 18 ms (5 and 25 ms), t-LTD is evident at P13–P21 and lost at P22–P30, both at the 5 and 25 ms intervals, as occurred with an interval of 18 ms: (*A*) P13–P21 t = 5 ms; (*B*) P22–P30, *t* = 5 ms; (*C*) P13–P21, *t* = 25 ms; (*D*) P22–P30, *t* = 25 ms.

Changes in the timing between presynaptic and postsynaptic activity during maturation could provoke the loss of plasticity during development. To test this hypothesis, we performed experiments using different timings between presynaptic and postsynaptic activity as a protocol to induce t-LTD, ranging from 5 to 25 ms. At both 5 and 25 ms, t-LTD was evident at P13–P21 and lost at P22–P30, as occurred when the interval was 18 ms. Moreover, with a 5 or 25 ms post–pre pairing, strong t-LTD was observed at P13–P21 (5 ms, 73 ± 6%, *n* = 7; 25 ms, 72 ± 7%, *n* = 6), whereas t-LTD was lost at P22–P30 in both cases (5 ms 98 ± 6%, *n* = 7; 25 ms 99 ± 7%, *n* = 6: Fig. 4).

### GABAergic Inhibition Does not Mediate the Loss of t-LTD During Development

GABAergic inhibition is an important regulator of plasticity (Paulsen and Moser 1998) and it augments as development proceeds (Banks et al. 2002). Thus, GABAergic inhibition is involved in the developmental changes of LTP induction in the hippocampus (Meredith et al. 2003) and it may mediate the closure of the window of t-LTD in the visual cortex (Hensch 2005). To assess whether enhanced GABAergic inhibition could account for the developmental change in the induction of t-LTD (t-LTD loss at P22–P30), we repeated the t-LTD experiments at P22–P30 in the presence of the GABA_A_ receptor antagonist bicuculline (10 μM). We found that t-LTD was still lost under these experimental conditions (104 ± 8%, *n* = 11: Fig. 6*A*,*B*), indicating that the increase in GABAergic inhibition does not induce a loss of t-LTD at CA3–CA1 synapses in the fourth week of development. Similarly, the presence of bicuculline (10 μM) did not affect t-LTD at P13–P21 (69 ± 7%, *n* = 7: Supplementary Fig. S4A).

### The Developmental Loss of t-LTD Involves Enhanced Inhibition Mediated by the Activation of Adenosine A_1_ Type Receptors

**Figure 5.**
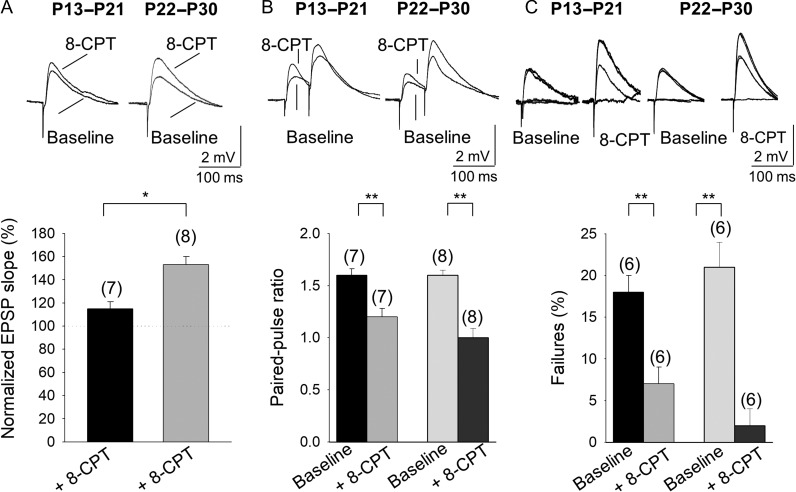
Presynaptic A_1_R-mediated inhibition increases with maturation. (*A*) 8-CPT affects the evoked EPSP slope at P13–P21 and P22–P30. (*B*) Effect of 8-CPT on the paired-pulse ratio (PPR). Note that 8-CPT produces a decrease in the PPR at P13–P21 and P22–P30. (*C*) The effect of 8-CPT on the number of failures of synaptic transmission. Note that 8-CPT produces a decrease in the number of failures. Error bars reflect the S.E.M. and the number of slices is shown in parentheses: *P < 0.05; **P < 0.01, unpaired Student’s *t*-test.

Adenosine participates in the gating of synaptic plasticity in the adult hippocampus (Arai et al. 1990; de Mendonça and Ribeiro 1994; Rex et al. 2005; zur Nedden et al. 2011), yet it is unknown if it mediates the loss of plasticity during development. It has been reported that the concentration of extracellular adenosine increases during development (Sebastiao et al. 2000; Rex et al. 2005; Kerr et al. 2013), and this increase in adenosine may affect synaptic efficacy and t-LTD. To confirm whether enhanced inhibition mediated by A_1_R activation occurs as the hippocampus matures, we determined the effect of the A_1_R antagonist 8-cyclopentyl-1,3-dimethylxanthine (8-CPT, 2 μM) on the EPSP slope at P13–P21 and P22–P30. This antagonist produced a small increase in the EPSP slope at P13–P21 (115 ± 6%, *n* = 7) and a robust effect at P22–P30 (153 ± 7%, *n* = 8: Fig. 5*A*), indicating that the activation of A_1_Rs mediates an increase in inhibition as development proceeds.

**Figure 6.**
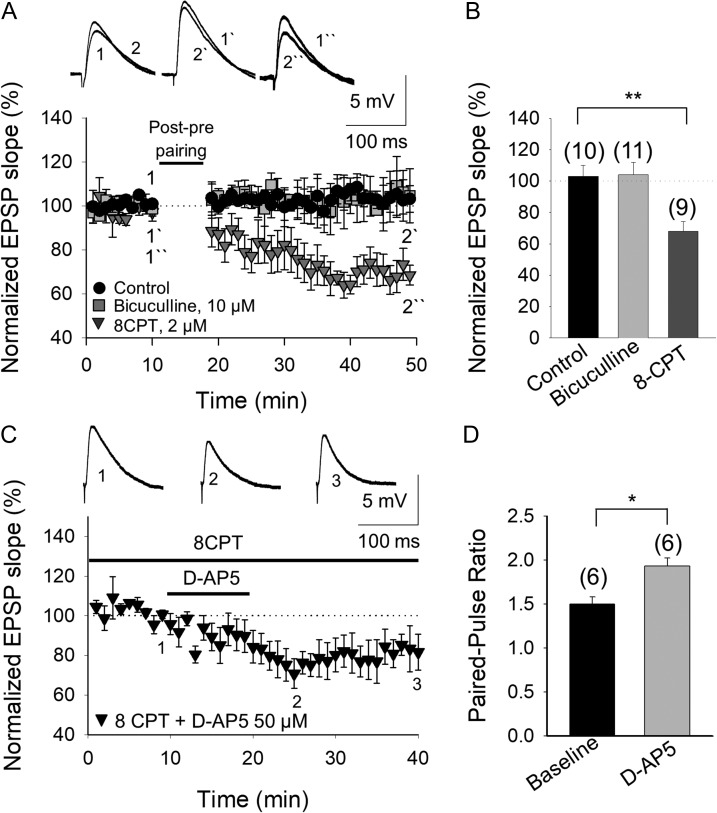
The developmental loss of t-LTD involves an increase in the inhibition mediated by adenosine A_1_ type receptor activation. (*A*) The loss of t-LTD is due to the activation of A_1_Rs and not to the activation of GABA_A_ receptors. The t-LTD lost at P22–P30 was not recovered in the presence of bicuculline (grey squares), whereas the lost t-LTD is completely recovered in the presence of the A_1_R antagonist 8-CPT (2 μM, grey triangles). The insets show the EPSP before (1, 1´, 1´´) and after (2, 2´, 2´´) post–pre pairing in control conditions (black circles), in the presence of bicuculline or 8-CPT. (B) Summary of the results. (C) The tonic activation of pre-NMDARs that is lost at P22–P30 is completely recovered when A_1_Rs are antagonized. In the presence of 8-CPT and with the postsynaptic neuron loaded with MK-801, D-AP5 induces a reversible decrease in the EPSP slope. Traces show the EPSP before (1), during (2), and after (3) exposure to D-AP5. (D) The paired-pulse ratio increases in the presence of D-AP5. The error bars indicate the S.E.M. and the number of slices is shown in parentheses: *P < 0.05; **P < 0.01, unpaired Student’s *t*-test.

By activating presynaptic A_1_Rs, adenosine exerts a potent inhibitory effect on glutamatergic synaptic transmission (for review see Dunwiddie and Masino 2001). Thus, the loss of tonic pre-NMDAR activation and the ensuing modulation of glutamate release at P22–P30 might be due to stronger inhibition of glutamate release mediated by presynaptic A_1_R activation. First, we confirmed that the effects observed were mediated by presynaptic A_1_Rs and for this, we analyzed the PPR and the number of failures in hippocampal slices treated with 8-CPT (2 μM). A decrease in the PPR was observed at both P13–P21 (1.6 ± 0.06% baseline, 1.2 ± 0.08 in 8-CPT, *n* = 7) and P22–P30 (1.58 ± 0.05% baseline, 1.0 ± 0.09 in 8-CPT, *n* = 8: Fig. 5*B*), indicative of a presynaptic effect. Failures in synaptic transmission were observed in several experiments and when we analyzed whether a change in the number of failures occurred with 8-CPT, we detected fewer failures in the presence of this antagonist at both P13–P21 (18 ± 2% baseline, 7 ± 2% in 8-CPT, *n* = 6) and P22–P30 (21 ± 3% baseline, 2 ± 2% in 8-CPT, *n* = 6: Fig. 5*C*). This effect was further evidence of a presynaptic mechanism and that adenosine activated A_1_Rs at a presynaptic locus. We then determined whether this increase in inhibition during development affected t-LTD, studying hippocampal slices from P22–P30 mice that lack t-LTD. A post-before-pre protocol induced t-LTD (68 ± 6%, *n* = 9 vs. 103 ± 7%, *n* = 10 in interleaved control slices) in the presence of 8-CPT (2 μM, Fig. 6*A*,*B*), indicating that the lack of t-LTD we observed was provoked by increased inhibition mediated by A_1_R activation. In the presence of 8-CPT (2 μM), t-LTD was not affected at P13–P21 (74 ± 9%, *n* = 6, Supplementary Fig. S4B).

We then examined if the enhanced inhibition mediated by A_1_Rs that affected glutamate release and prevented t-LTD, also affected the tonic activation of pre-NMDARs. As such, we assessed the effect of antagonizing the activity of adenosine mediated by A_1_Rs with 8-CPT in hippocampal slices from P22 to P30 mice. With postsynaptic NMDARs blocked by loading the postsynaptic cell with MK-801, the tonic activation of pre-NMDARs was recovered in the presence of 8-CPT and the EPSP slope decreased (76 ± 7%, *n* = 6, vs. 102 ± 6%, *n* = 5 in interleaved slices not treated with 8-CPT: Fig. 6*C*,*D*), reaching a similar level to that observed in untreated slices at P13–P21 (Fig. 2). In addition, the increased PPR observed at P13–21 was also recovered under these experimental conditions (Fig. 6*D*). Hence, the activation of presynaptic A_1_Rs by adenosine appears to dampen glutamate release and prevent the induction of t-LTD, at the same time preventing tonic activation of pre-NMDARs. These results indicate that a high probability of glutamate release (enough to tonically activate pre-NMDARs) is necessary for t-LTD and as such, tonic activation of pre-NMDARs is lost when this probability of release decreases, which is followed by a loss of t-LTD.

### A_1_R Activation at P13–P21 Closes the Window of Plasticity

**Figure 7.**
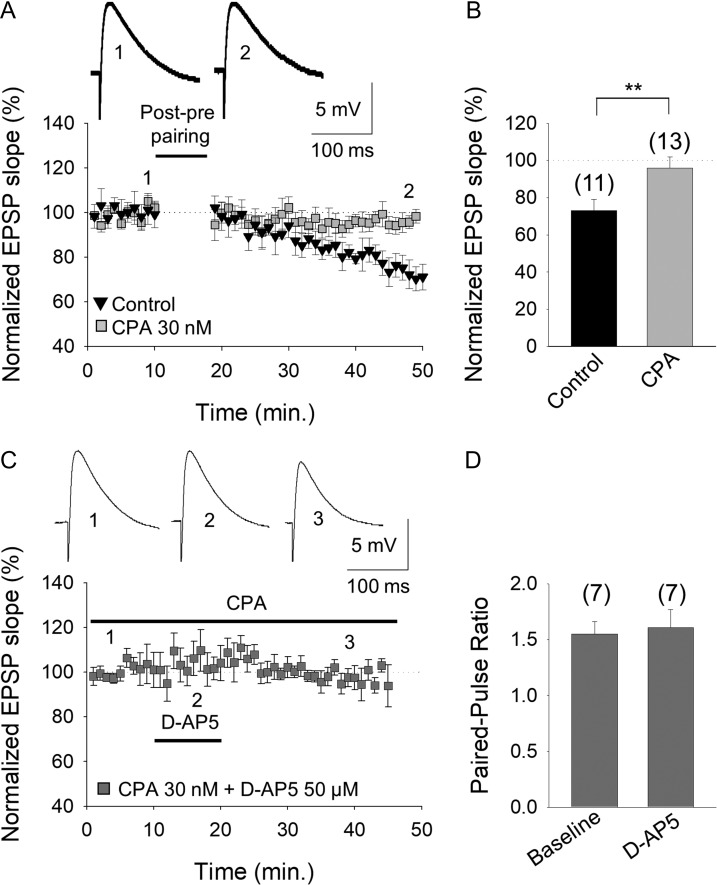
An increase in A_1_R mediated-inhibition closes the window of plasticity for t-LTD at P13–P21. (*A*) Activation of A_1_Rs at P13–P21 by the agonist CPA prevented the induction of t-LTD. The evoked EPSP slopes monitored in control slices (black triangles) and in slices treated with the A_1_R agonist CPA (grey squares) following a post–pre pairing are shown. The traces show the EPSP before (1, 1´) and 30 min after (2, 2´) pairing in control slices, and in slices treated with CPA. (*B*) Summary of the results. (*C*) Tonic pre-NMDAR activation at P13–P21 is lost in the presence of the A_1_R agonist CPA. D-AP5 did not affect the evoked EPSP slope in the presence of CPA. The traces show the EPSP before 1), during 2), and after 3) exposure to D-AP5. (*D*) The paired-pulse ratio was not affected by D-AP5 in the presence of CPA (with the postsynaptic cell loaded with MK-801). The error bars represent the S.E.M. and the number of slices is shown in parentheses: **P < 0.01, unpaired Student’s *t*-test.

If higher extracellular adenosine concentrations during development more strongly activate presynaptic A_1_Rs at CA3–CA1 hippocampal synapses, provoking the loss of t-LTD at P22–P30, it may be possible to close the window of plasticity windows earlier in the development by enhancing A_1_R activation (e.g., at P13–P21 when t-LTD is robust). Indeed, the induction of t-LTD was in fact impaired when hippocampal slices from P13 to P21 animals were maintained for 1 h in the presence of the A_1_R agonist (2R, 3R, 4S, 5R)-2-(6-(cyclopentylamino)-9H-purin-9-yl)-5-(hydroxymethyl)tetrahydrofuran-3,4-diol, N6-Cyclopentyl-adenosine (CPA, 30 nM; 96 ± 6%, *n* = 13, vs. t-LTD observed in interleaved slices 73 ± 6%, *n* = 10: Fig. 7*A*,*B*). Exposure to CPA per se decreased the EPSP slope (to 53 ± 10% of baseline, *n* = 6) and it increased the PPR (1.5 ± 0.1 baseline; 2 ± 0.2 in CPA, *n* = 5), again consistent with a presynaptic site of action (Supplementary Fig. S5). If A_1_R activation decreased the probability of glutamate release, mediating the loss of tonic pre-NMDAR activation, and if tonic pre-NMDAR activation is directly correlated with the induction of t-LTD, D-AP5 would not be expected to affect the EPSP slope after exposure to CPA. In the presence of MK-801 in the postsynaptic neuron, there was indeed no tonic pre-NMDAR activation in P13–P21 hippocampal slices exposed to CPA (30 nM; 98 ± 4%, *n* = 7 vs. tonic activation in interleaved slices 78 ± 8%, *n* = 6: Fig. 7*C*). Moreover, there was no change in the PPR in these experimental conditions after exposure to D-AP5 (1.5 ± 0.2% baseline; 1.6 ± 0.2% after D-AP5 *n* = 6: Fig. 7*D*) consistent with stabilization of the probability of glutamate release.

These results indicate that during development, presynaptic A_1_R-mediated inhibition is crucial for the plastic properties of these synapses. Moreover, A_1_R activation modulates the probability of glutamate release and hence, tonic pre-NMDAR activation and the induction of t-LTD.

### The Developmental Loss of t-LTD Requires Adenosine From Astrocytes

The role of adenosine in activating presynaptic A_1_Rs and driving the developmental loss of t-LTD in the hippocampus raises interest in the source of this nucleoside. In neurons, stimulated adenosine release seems to occur through equilibrative nucleoside transporters (ENTS) and it is known that an important part of the released adenosine in the nervous system arises from the extracellular metabolism of ATP released by astrocytes (Dunwiddie and Masino 2001; Wall and Dale 2013). Astrocyte activation may provoke glutamate and other gliotransmitter release, such as d-serine (Andrade-Talavera et al. 2016), ATP or adenosine (Araque et al. 2014). We investigated the possible involvement of astrocytes in the adenosine release mediating presynaptic A_1_R activation, and in the subsequent loss of tonic pre-NMDAR activation and t-LTD during development.

**Figure 8.**
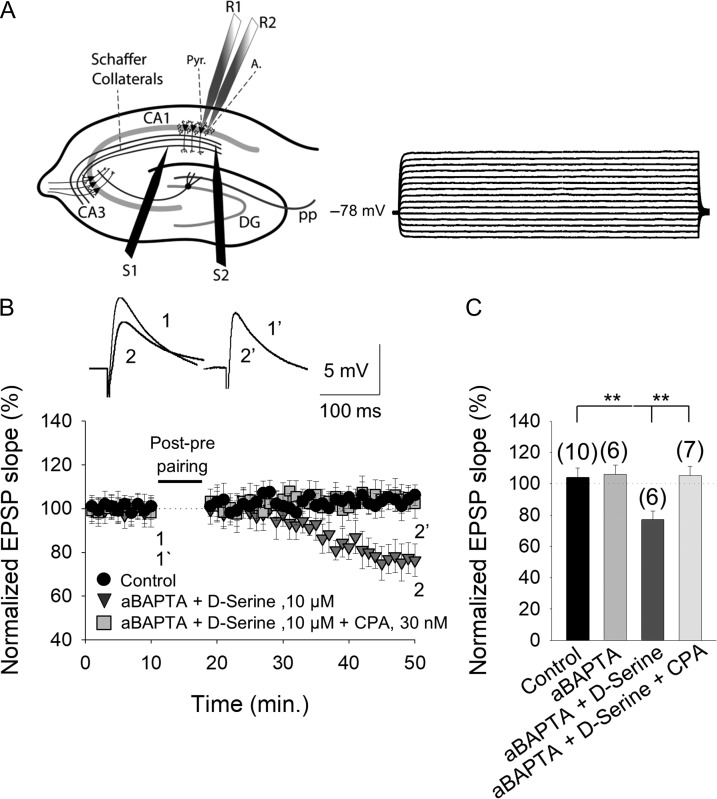
The adenosine involved in preventing t-LTD at P22–P30 is from astrocytes. (*A*) Left, scheme showing the general experimental set-up: R1 and R2, recording electrodes; S1 and S2, stimulating electrodes; Pyr, pyramidal neuron; A, astrocyte; right, voltage responses of an astrocyte shown in current-clamp. (*B*) In astrocyte-neuron dual recordings, with the calcium chelator BAPTA injected into the astrocyte via the recording pipette (aBAPTA), and with D-serine (100 μM) added to the bath, a post–pre pairing protocol induced t-LTD (grey triangles) but not in control conditions (no BAPTA and no d-serine, black circles). The presence of CPA impaired the t-LTD observed with aBAPTA and d-serine (dark grey squares). Inset: representative traces at the baseline (1 and 1´) and 30 min after the pairing protocol (2 and 2´) in the presence of aBAPTA and d-serine alone, with or without CPA. (*C*) Summary of the results, with the error bars reflecting the S.E.M. and the number of slices shown in parentheses: **P < 0.01, unpaired Student’s *t*-test.

If ATP/adenosine from astrocytes is involved in the loss of t-LTD during development and this ATP/adenosine is released in vesicles, impeding vesicular release from astrocytes will prevent the effects of adenosine and permit t-LTD induction. Using a patch pipette, individual astrocytes were loaded with the Ca^2+^ chelator BAPTA (20 mM, Fig. 8*A*) and pyramidal neurons were recorded 15–30 min. after BAPTA loading, this chelator inhibiting vesicle and Ca^2+^-dependent gliotransmitter release from these astrocytes (Parpura and Zorec 2010). BAPTA loading of astrocytes (aBAPTA) and the addition of d-serine (recently shown to be necessary for the induction of this form of t-LTD (Andrade-Talavera et al. 2016) permitted t-LTD induction in CA1 pyramidal neurons in the proximity (50–100 μm) from the BAPTA-loaded astrocyte (78 ± 5%, *n* = 6: Fig. 7*B*,*C*), in contrast to the typical loss of t-LTD at CA3–CA1 synapses at P22–P30 (111 ± 10%, *n* = 6: Figs 1*F* and 8*B*,*C*). However, t-LTD induction was impeded in neurons proximal to BAPTA-loaded astrocytes in the presence of D-serine and the A_1_R agonist, CPA (102 ± 7%, *n* = 6: Fig. 8*B*,*C*). At P22–P30, when astrocytes were not loaded with BAPTA and the slices were treated with the CB_1_R antagonist AM251 (3 μM), t-LTD was not recovered in the presence of 8-CPT (102 ± 8%, *n* = 5). In astrocytes loaded with BAPTA alone, t-LTD was prevented (105 ± 7%, *n* = 6: Fig. 8*C*, Supplementary Fig. S6). Together, these results indicate that a gliotransmitter from astrocytes (adenosine or its precursor, ATP) and CB_1_Rs are responsible for the lack of t-LTD at P22–P30.

To gain more insight into the role of astrocytes in the release of the ATP/adenosine gliotransmitter affecting presynaptic glutamate release, we performed dual recordings in astrocytes and neighboring pyramidal neurons, monitoring the time course of the EPSP slope evoked by basal stimulation at 0.2 Hz at P13–P21 and P22–P30. At P13–P21, direct stimulation of astrocytes (depolarization from −80 to 0 mV at 0.4 Hz for 10 min) produced a clear decrease in the eEPSP slope (76 ± 9%, *n* = 6: Supplementary Fig. S8) that was not evident in the presence of 8-CPT (2 μM; 106 ± 5%, *n* = 6: Supplementary Fig. S7). A decrease in the EPSP slope was observed at P22–P30 when astrocytes were stimulated (81 ± 5%, *n* = 6: Supplementary Fig. S8) but again, astrocyte stimulation did not affect EPSP slope in the presence of 8-CPT (107 ± 7%, *n* = 6: Supplementary Fig. S7).

Together, these results indicate that the release of ATP/adenosine by astrocytes alters the probability of glutamate release (via activation of presynaptic A_1_Rs), influencing the loss of t-LTD during development and closing the window of plasticity.

## Discussion

### Loss of t-LTD by the Fourth Week of Development

We studied synaptic plasticity during development, from P8 to P21, using a post–pre protocol that induced robust presynaptic t-LTD. Such t-LTD was not evident in CA3–CA1 synapses after ~P21, consistent with earlier reports that the capacity for synaptic depression in cortical synapses declines with age (Bear and Abraham 1996; Corlew et al. 2007; Banerjee et al. 2009; Rodríguez-Moreno et al. 2013). These results extend the developmental period of timing-dependent LTD reported previously for CA3–CA1 synapses in the hippocampus into young adulthood, and they extend the developmental loss of t-LTD observed in other brain regions to the hippocampus.

### Pre-NMDARs are Tonically Active, and They Enhance Evoked and Spontaneous Glutamate Release at P13–P21 but not at P22–P30

There is evidence that pre-NMDARs physiologically modulate transmitter release by acting as autoreceptors (Mameli et al. 2005; Jourdain et al. 2007; McGuinness et al. 2010, Bouvier et al. 2018). Similarly, D-AP5 decreases glutamate release at P13–P21 when postsynaptic NMDARs are blocked, confirming the tonic activation of pre-NMDARs in the hippocampus and their role as autoreceptors at SC–CA1 synapses. Tonic activation and the modulation of glutamate release are lost as development proceeds, indicating a reduction in the probability of release, as observed previously in the somatosensory (Frick et al. 2007), auditory (Oswald and Reyes 2008), visual (Cheetham and Fox 2010), and prefrontal (González-Burgos et al. 2008) cortices. Pre-NMDARs are tonically active during development in the entorhinal (Berretta and Jones 1996) visual (Corlew et al. 2007), somatosensory (Brasier and Feldman 2008) cortices, and indirectly in the hippocampus (Mameli et al. 2005). The results presented here confirm that hippocampal pre-NMDARs are tonically active at P13–P21, coincident with a critical period of plasticity, whereas this tonic activation is completely lost when this plasticity ends at P22–P30, suggestive of a direct relationship between these two phenomena. We demonstrated this relationship by manipulating the probability of glutamate release through changes in the extracellular Ca^2+^ concentration, which directly affects plasticity and the plasticity windows. Hence, t-LTD can be prevented at P13–P21 by decreasing Ca^2+^ in the extracellular fluid and the loss t-LTD recovered at P22–P30 by increasing this Ca^2+^ concentration.

Pre-NMDARs may be required to maintain high probabilities of glutamate release, as generally observed during early development. This high probability of transmitter release may be necessary for terminals to establish connections with postsynaptic cells and to maintain neurotransmission when such postsynaptic neurons are still not completely developed (Rumpel et al. 2004). The loss of tonic pre-NMDAR activation correlates with the loss of t-LTD, as indicated above, suggesting that pre-NMDARs need to be activated by ambient glutamate to induce t-LTD. Our data clearly show that pre-NMDARs can be activated by evoked and spontaneous glutamate release, indicating that ambient glutamate activates pre-NMDARs at these synapses during early developmental stages.

The exact presynaptic intracellular mechanism by which the activation of pre-NMDARs can increase glutamate release when these receptors are tonically active and at the same time may mediate t-LTD as a decrease in glutamate release that is long-lasting is not currently known. Likewise, it is unclear what happens intracellularly at the presynaptic side to mediate the t-LTD loss when those receptors are not tonically active. Whether these effects are mediated by distinct pre-NMDARs or by the same type of NMDARs activating different intracellular cascades is not clear at present. Calcineurin is required for pre-NMDAR activation to mediate t-LTD, as recently demonstrated elsewhere (Andrade-Talavera et al. 2016). While the mechanisms by which calcineurin mediates t-LTD are not known, several presynaptic proteins might be involved, such as those involved in exocytosis, endocytosis and in the regulation of the size of releasable, recycling and reserve pools of synaptic vesicles (Leenders and Sheng 2005; Kim and Ryan 2010), as well as presynaptic calcium channels (Kaeser and Südhof 2005; Kim and Ryan 2013) and their interaction with the release machinery. Further experiments should determine the exact mechanisms underlying these phenomena.

### Pre-NMDARs are Present at Schaffer Collaterals in the Hippocampus and the Number of Receptors Decreases as Development Proceeds

We present anatomical evidence that pre-NMDARs are present at SCs early in development (P15) and that their prevalence falls 52% in the following 15 days (at P30). The decrease in the number of pre-NMDARs correlates with the loss of t-LTD, representing a possible explanation for this developmental change, although this result does not explain the loss of tonic pre-NMDAR activation. As pre-NMDARs do not disappear completely during development, other additional factors must explain the loss of plasticity observed. It is possible that the number of pre-NMDARs falls when these receptors are not tonically activated and that the pre-NMDARs remaining at P22–P30 do not participate in t-LTD or are insufficient to drive t-LTD. As such, the role of these remaining pre-NMDARs at these synapses at P22–P30 merits further study. One possibility is that these receptors, while not tonically active, act as autoreceptors and modulate glutamate release, as occurs in the visual cortex (Corlew et al. 2007).

Our data are consistent with changes in pre-NMDAR function during maturation. Indeed, the developmental loss of pre-NMDARs may be a more general feature as a similar change in pre-NMDAR function occurs in L5 of the rat entorhinal cortex, where pre-NMDARs are very active at 5 weeks and much less so after 5 months (Yang et al. 2006). A similar developmental loss of pre-NMDARs has also been described in the visual cortex, where these receptors facilitate spontaneous release at *P* < 20 but not at *P* > 23 (Corlew et al. 2007). It is also possible that only some subsets of terminals with pre-NMDARs participate in t-LTD and that the reduced number observed here may reflect those directly involved in t-LTD (Buchanan et al. 2012). Our EM data demonstrate that NMDARs are present at presynaptic sites. The subcellular physiological relevance of pre-NMDARs for t-LTD will be addressed by future studies administering caged MK-801 to presynaptic neurons and obtaining paired recordings between synaptically connected CA3 and CA1 neurons, as achieved at L4–L2/3 synapses in the somatosensory cortex (Rodríguez-Moreno et al. 2011; Reeve et al. 2012).

### The Loss of t-LTD is not Due to a Shift in the Coincidence Time Window Needed to Induce t-LTD

We checked the possibility that a shift in the time windows of plasticity could explain the loss of t-LTD observed. We observed the same phenomenon when different timings between presynaptic and postsynaptic activity were used in protocols to induce t-LTD (5 and 25 ms), such that t-LTD disappeared at P22–P30. While we did not check other intervals, these results strongly suggest that a shift in the time windows does not mediate the loss of t-LTD observed with maturation.

### Adenosine A_1_ Type Receptors Mediate the Enhanced Inhibition That is Responsible for the Loss of Tonic Activation and of Plasticity During Development

We clearly show here that enhanced inhibition is crucial to close the window of plasticity. This enhanced inhibition is not mediated by GABA_A_ receptor activation as the loss of t-LTD at the fourth week of development is not affected by the presence of bicuculline. By contrast, an increase in inhibition mediated by A_1_R activation does appear to be involved in the loss of tonic pre-NMDAR activation and in t-LTD. In the presence of an A_1_R antagonist, t-LTD does not disappear during development, a clear indication of the crucial role of adenosine in t-LTD. Indeed, antagonizing A_1_Rs at P22–P30 recovers the tonic activation of pre-NMDARs and enhanced glutamate release, suggesting a direct correlation between tonic pre-NMDAR activation and the presence of t-LTD. To more directly demonstrate the involvement of A_1_Rs in the tonic activation of pre-NMDARs and t-LTD, reciprocal experiments were performed. Thus, activating A_1_Rs at P13–P21, when a robust t-LTD can be induced and pre-NMDARs are tonically activated (modulating glutamate release), prematurely closed the window of plasticity and induced the loss of t-LTD, as observed in older mice (P22–P30). Likewise, agonists of A_1_Rs provoke a loss of tonic pre-NMDAR activation, again relating this effect to the presence of t-LTD. Accordingly, we have identified the mechanism underlying this closure of the window of plasticity, opening the way to the pharmacological manipulation of plasticity and of the t-LTD, which is likely to be relevant to understand brain function and in health.

The data presented indicate that presynaptic A_1_R activation drives a decrease in glutamate release, the most parsimonious explanation for the loss of tonic activation and the loss of t-LTD observed. Indeed, adenosine exerts its potent inhibitory effect by modulating the glutamatergic synaptic transmission mediated through presynaptic A1R activation (Dunwiddie and Masino 2001). We used 2 different approaches to determine the site of action of adenosine and A_1_Rs. Fluctuation analysis and PPR were consistent with presynaptic changes, suggesting a presynaptic site of action for the adenosine antagonist and agonist used, and indicating presynaptic activity of A_1_Rs. The concentration of extracellular adenosine increases during development (Sebastiao et al. 2000; Rex et al. 2005; Kerr et al. 2013), as reflected by the stronger increase in the EPSP slope induced by 8-CPT in the hippocampus at P22–P30 than at P13–P21. Hence, we propose that the activation of presynaptic A_1_Rs increases during development due to the increase in the amount of extracellular adenosine. This in turn inhibits glutamate release, reducing the ambient glutamate and impairing the tonic activation of pre-NMDARs, which causes a loss of the t-LTD. Thus, this increase in adenosine seems to alter glutamate release, tonic activation of pre-NMDARs, synaptic efficacy and t-LTD. Whether this is also the cause of the observed decrease in the number of pre-NMDARs remains to be determined. NMDARs may be responsible for closing this window of plasticity in the visual cortex (Corlew et al. 2007), although it may be possible that the decrease in the number of pre-NMDARs is a consequence and not a cause of the loss of plasticity, as suggested by our present results, particularly as tonic activation is recovered in the presence of an A_1_R antagonist at P22–P30 (when it is naturally lost).

### Astrocytes are Required for the Loss of Plasticity During Development

An important part of the adenosine released comes from the extracellular metabolism of ATP released by astrocytes (for review, see Dunwiddie and Masino 2001; Wall and Dale 2013). Identifying the source of adenosine is complex as it may be released directly from neurons by exocytosis (Klyuch et al. 2012) or via transporters (Lovatt et al. 2012). Adenosine may also be released indirectly following the rapid extracellular metabolism of the ATP released exocytotically by neurons (Jo and Sclichter 1999) or glial cells (Pascual et al. 2005), or through glial gap junction hemichannels (Huckstepp et al. 2010), as well as by other mechanism that may act simultaneously. We assessed the possible astrocytic source of the adenosine that activates A_1_Rs, as these cells have been described as a source of adenosine (mostly in the form of the adenosine precursor, ATP) and other gliotransmitters released after the calcium mobilization of astrocytic vesicles (Araque et al. 2014). At P22–P30, BAPTA loading of astrocytes recovers the t-LTD and this recovery was blocked in the presence of CPA, demonstrating that adenosine of astrocytic origin is required for the loss of t-LTD. However, while we prove that there is a requirement for astrocytes to provide adenosine, other postsynaptic cells or interneurons may also contribute adenosine to this process (Manzoni et al. 1994). We also demonstrate that directly stimulating astrocytes increases the extracellular adenosine levels sufficiently to affect the EPSP slope, thereby affecting glutamate release.

**Figure 9.**
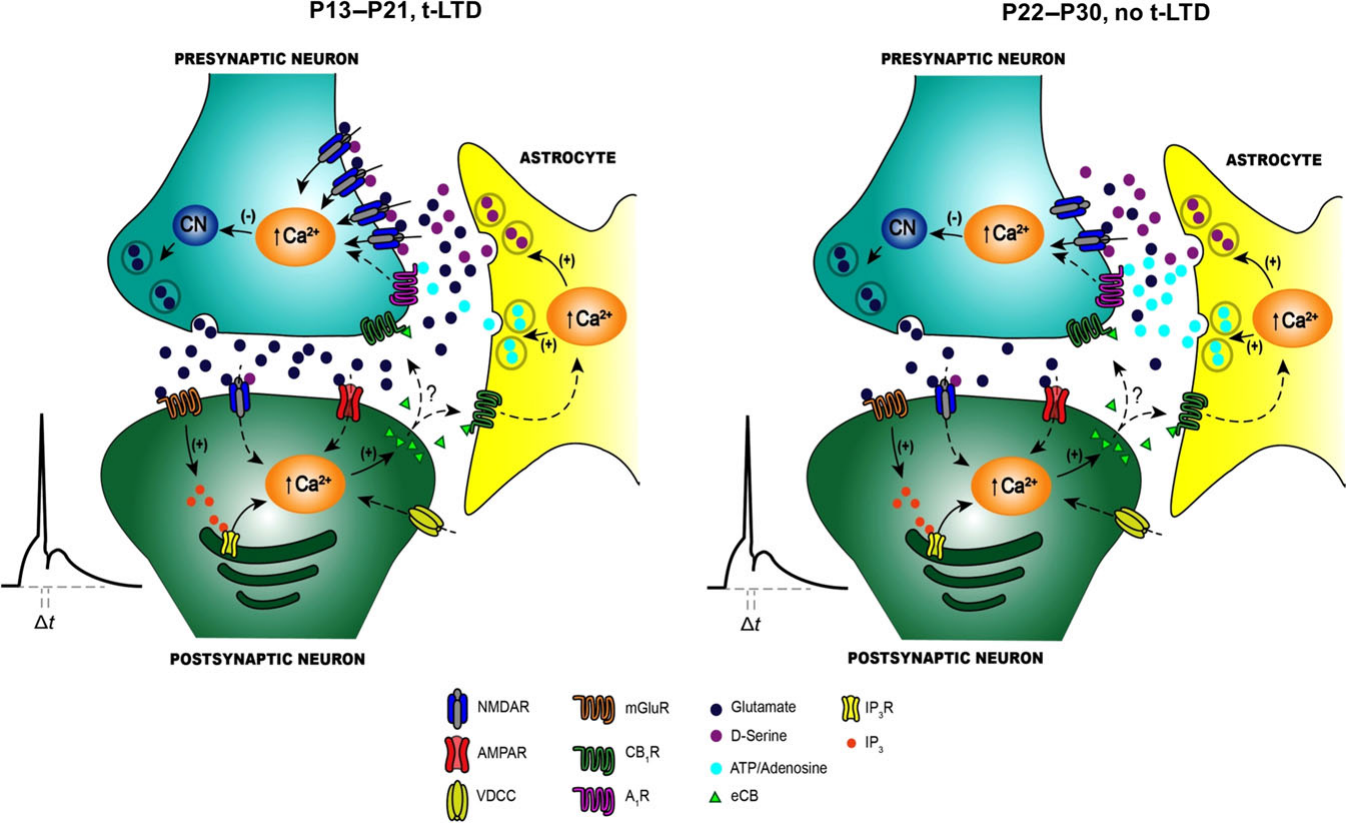
Scheme of the differences in signaling between early (P13–P21) and late (P22–P30) stages of development. (A) At P13–P21, t-LTD is induced by a post–pre single-spike pairing protocol. Postsynaptic action potentials activate voltage-dependent Ca^2+^ channels (VDCCs) and the presynaptically released glutamate activates postsynaptic mGlu5 receptors. These receptors synergistically activate PLC and produce IP_3_, provoking Ca^2+^ release from internal stores and DAG production, which serves as a precursor for endocannabinoid (eCB) synthesis. The eCB signal activates CB1 receptors, facilitating d-serine release from astrocytes. Together with the glutamate released from presynaptic neurons, this d-serine activates presynaptic NMDARs on Schaffer Collateral boutons. This leads to an increase in presynaptic Ca^2+^, calcineurin activation and synaptic depression. (B) At P22–P30, t-LTD does not develop and the main differences at these synapses at the 2 stages of development are: a change in the probability of glutamate release (higher at P13–P21 than at P22–P30), the tonic activation of pre-NMDARs at P13–P21 but not at P22–P30, a decrease in the number of pre-NMDARs at P22–P30, and an increase in adenosine release from astrocytes at P22–P30 compared with P13–21.

Whether the increase in extracellular adenosine with maturation is due to an increase in the number of astrocytes or to increased release, or whether other components that participate in the induction of this form of t-LTD are altered by maturation will merit further study. For the moment we know that D-serine is necessary to recover t-LTD at P22–P30 when A_1_Rs are antagonized and that in the absence of D-serine, 8-CPT does not rescue t-LTD. CB_1_Rs also affect the rescue of t-LTD at P22–P30 and when CB_1_Rs are antagonized at this age, the addition of 8-CPT does not recover t-LTD. Thus, as all the recently described postsynaptic mechanisms are functional at P22–P30 (postsynaptic mGluRs, postsynaptic calcium, release of eCB, activation of CB_1_Rs: Andrade-Talavera et al. 2016), the amount of extracellular adenosine at P22–P30 is higher and sufficient to strongly dampen presynaptic glutamate release by activating presynaptic A_1_Rs, which will influence pre-NMDAR activation and t-LTD (Fig. 9).

### What is the Physiological Role of this Form of Plasticity?

The true influence of STDP in the hippocampus remains unclear and further studies are necessary to determine the specific role of t-LTD. The form of LTD described here is only evident until the third week of development, indicating its relevance during development and possibly, in the refinement of synapses. Indeed, LTD is thought to play an important role in plasticity during development (Buonomano and Merzenich 1998; Feldman and Brecht 2005) and LTD is thought to weaken excitatory synapses that are underused or behaviorally irrelevant.

While for the moment data indicating a direct correlation between the observed t-LTD and morphological changes at presynaptic terminals and of dendrites do not exist, future studies will unequivocally determine the role of this form of t-LTD in the refinement of synapses during development. In this sense, different changes occur in dendritic refinement as the hippocampus matures. For instance, there is a change in spine type at P30 compared with P15, specifically a decrease in the presence of mushroom spines (Supplementary Fig. S8). This would suggest there are changes in the density of this spine type during maturation (the most stable of spines), consistent with the elimination of bad or useless synaptic connections through LTD (Mel et al. 2017). Experiments measuring changes in dendrites and axon terminals while studying plasticity will directly indicate the exact role of this form of t-LTD, and the morphological significance of the closing of this window of plasticity.

In summary (Fig. 8), our results indicate that t-LTD is present at CA3–CA1 synapses of the mouse hippocampus until the third week of development and that it disappears in the fourth week. The disappearance of this form of t-LTD due to enhanced inhibition during maturation is driven by the activation of presynaptic A_1_Rs by adenosine of astrocytic origin. These events mediate a decrease in the release probability of glutamate and the loss of tonic pre-NMDAR activation. These results provide direct evidence of a mechanism responsible for closing a window of plasticity during development and provide a possible means to directly manipulate a window of plasticity for t-LTD. This mechanism may be involved in synaptic remodeling during development and it may also be relevant for brain repair, sensory recovery and the treatment neurodevelopmental disorders.

## Supplementary Material

Supplementary_Figures_and_Methods_R1_bhy194

## Notes

We thank Dr Cristina Calvino for her technical assistance and Dr Mark Sefton for linguistic assistance. *Conflict of Interest*: the authors have no competing financial interests to declare.
