# Supplementary material for: Adenosine Receptor-Mediated Developmental Loss of Spike Timing-Dependent Depression in the Hippocampus
Source: Cereb Cortex. 2018 Aug 31;29(8):3266–81. doi: 10.1093/cercor/bhy194 (PMC6644873; doi:10.1093/cercor/bhy194)
Supplement: Supplementary_Figures_and_Methods_R1_bhy194 [file supplementary_figures_and_methods_r1_bhy194.docx]

**Adenosine receptor-mediated developmental loss of spike timing-dependent depression in the hippocampus**

Mikel Pérez-Rodríguez, Luis E Arroyo-García, José Prius-Mengual, Yuniesky Andrade-Talavera, José A Armengol, Eva M Pérez-Villegas, Paloma Duque-Feria, Gonzalo Flores and Antonio Rodríguez-Moreno

**Supplementary Figures and Methods**

**Supplementary Figure 1. MK-801, added extracellularly or loaded into the postsynaptic neuron, completely blocks NMDAR-mediated currents.** (A) The postsynaptic NMDA current was monitored during voltage-clamp experiments at +40 mV, and it was isolated by adding bicuculline (10 µM) and NBQX (10 µM) to the superfusion fluid. After stable recording of the NMDAR current for 10 min., MK-801 was added to the bath (eMK-801) and it produced a gradual decrease in the amplitude of the NMDAR-mediated current, completely blocking it after 8-10 min. (B) When MK-801 was loaded into the postsynaptic neuron, a gradual decrease of NMDAR-mediated currents was observed just 1-2 min. after the start of the stimulation, reaching a complete block after 8-10 min.

**Supplementary Figure 2. Glutamate transporters are not involved in the loss of t-LTD with maturation.** The presence (at P13-P21, A) or absence (at P22-P30, B) of tonic preNMDAR activation is not due to changes in glutamate transporters with maturation, given that the results obtained at P13-P21 and at P22-P30 were similar to control experiments in untreated slices in the presence of the glutamate transporter blocker TBOA (50 µM). **Changes in the probability of glutamate release affect t-LTD.** (C, D) The t-LTD is directly linked to tonic activation of preNMDARs by glutamate, as changing the probability of glutamate release directly affect the t-LTD. When the extracellular Ca^2+^ concentration is decreased to 1 mM, t-LTD cannot be induced at P13-P22 (C) whereas t-LTD is rescued at P22-P30 (when it is normally lost) when the calcium concentration is increased to 4 mM (D).

**Supplementary Figure 3. Effect of D-AP5 on the mEPSP frequency and amplitude at CA3-CA1 synapses at P13-P21 and P22-P30.** D-AP5 produces a decrease in the mEPSP frequency but it does not affect mEPSP amplitude at P13-P21 (A), whereas it affects neither mEPSP frequency nor amplitude at P22-P30 (B). Error bars are the S.E.M. and the number of slices is shown in parentheses: * p < 0.05, unpaired Student's *t*-test.

**Supplementary Figure 4.** At P13-P21, neither the presence of bicuculline (A) nor that of 8-CPT (2 µM: B), affected t-LTD.

**Supplementary Figure 5. The presynaptic effect of CPA at baseline.** (A) CPA (30 nM) produces a decrease in the EPSP slope at baseline. (B) CPA produces a statistically significant increase in the paired-pulse ratio. Error bars represent the S.E.M. and the number of slices is shown in parentheses: * p < 0.05, unpaired Student's *t*-test.

**Supplementary Figure 6.** At P22-P30, t-LTD was prevented when astrocytes were loaded with BAPTA alone.

**Supplementary Figure 7. Astrocyte stimulation affects the EPSP slope in neighboring pyramidal neurons at CA3-CA1 synapses.** Performing dual recordings in astrocytes and neighboring pyramidal neurons, and monitoring the slope of the EPSP evoked by basal stimulation at 0.2 Hz at P13-P21 and P22-P30, indicated a clear decrease in the slope of the eEPSP (76 ± 9%) when the astrocyte was directly stimulated at P13-P21 (depolarized from -80 mV to 0 mV at 0.4 Hz for 10 minutes: A). In slices treated with 8-CPT (2 µM), astrocyte stimulation did not affect the EPSP slope (A). At P22-P30, a decrease in the EPSP slope was observed (81 ± 5%) in untreated slices whereas in the presence of 8-CPT, no effect on the EPSP slope (107 ± 7%) was observed after stimulation of the astrocyte.

**Supplementary Figure 8. Maturation from P15 to P30 generates changes in the morphology of CA1 pyramidal neurons.** (A) From P15 to P30 there is a decrease in the mushroom type spines on neurons. Representative photomicrographs of Golgi-Cox stained pyramidal neurons from the CA1 hippocampus (40X) and a representative distal dendritic segment (100X) at P15 and P30. (B) Summary of the spine distribution. The error bars represent the S.E.M. (n = 6 mice): ****P < 0.0001 unpaired Student’s t-test.

**Supplementary Figure 8 Methods**

*Golgi-Cox stain method*

Mice aged P15 and P30 (n = 6 animals per group) were anesthetized deeply with sodium pentobarbital (75 mg/kg body weight, i.p.) and perfused intracardially with 0.9% saline solution. The brain of each mouse was removed and stained using the modified Golgi-Cox method, as described previously (Flores et al., 2005). Coronal hippocampal vibratome sections (200 µm thick: Campden Instrument, MA752, Leicester, UK) were collected on clean, gelatin-coated microscope slides and treated with ammonium hydroxide for 30 min, followed by 30 min in Kodak Film Fixer. The sections were finally rinsed with distilled water and mounted in a resinous medium (Robinson and Kolb, 1997; Tendilla-Beltran, 2016).

*Microscopic observation and Sholl analysis*

CA1 Pyramidal cells (plate 29-37 of the Paxinos and Watson Atlas, 1998) were selected for this study. For each animal, neurons from both the left and right hippocampal formation were drawn at a magnification of 250X using *camera lucida* (DMLS, Leica Microscope) by a trained observer who was blind to the experimental conditions. Pyramidal neurons were readily identified by their characteristic triangular soma, apical dendrites, basolateral dendrites and numerous dendritic spines. Sequential two-dimensional reconstructions of the entire dendritic tree were generated for each neuron and dendritic tracings were quantified through a Sholl analysis (Sholl, 1953). To calculate the spine density, a length of the dendrite (at least > 10 μm long) was traced (at 1,000X), the exact length of the dendritic segment was calculated and the number of spines along this stretch of dendrite was counted (spines/10 µm).

*Dendritic spine type analysis*

Dendritic spines from CA1 neurons stained using the Golgi-cox method were typified based on their structure, as described previously (Fiala, 2002; Bello-Medina, 2016): mushroom, thin, stubby, branched and no-classification. The spines were analyzed using a coupled device microscope (Leica manual magnification changer No.11505252) to obtain a 2000X magnification on a light microscope (DMLS, Leica Microscope), and 100 dendritic spines per neuron were typified, 6,000 dendritic spines from the CA1 region in the whole study.

**Supplementary References**

Bello-Medina PC, Flores G, Quirarte GL, McGaugh JL, Prado-Alcalá RA. 2016. Mushroom spine dynamics in medium spiny neurons of dorsal striatum associated with memory of moderate and intense training. Proc Natl Acad Sci USA. 113 (42): E6516-E6525.

Fiala JC, Spacek J, Harris KM. 2002.Dendritic spine pathology: cause or consequence of neurological disorders? Brain Res Rev. 39: 29-54.

Flores G, Alquicer G, Silva-Gómez AB, Zaldívar G, Stewart J, Quirion R, Srivastava LK. 2005. Alterations in dendritic morphology of prefrontal cortical and nucleus accumbens neurons in post-pubertal rats after neonatal excitotoxic lesions of the ventral hippocampus. Neuroscience. 133: 463-470.

Paxinos G, Watson C. 1998. The rat brain in stereotaxic coordinates. 4th Edition. Apnet Edtiorial: 474.

Robinson TE, Kolb B. 1997. Persistent structural modifications in nucleus accumbens and prefrontal cortex neurons produced by previous experience with amphetamine. J Neurosci. 17: 8491-8497.

Sholl DA. 1953. Dendritic organization in the neurons of the visual and motor cortices of the cat. J Anat. 87: 387-406.

Tendilla-Beltrán H, Arroyo-García LE, Díaz A, Camacho-Abrego I, de la Cruz F, Rodríguez-Moreno A, Flores G. 2016. The effects of amphetamine exposure on juvenile rats on the neuronal morphology of the limbic system at prepubertal, pubertal and postpubertal ages. J Chem Neuroanat. 77: 68-77.
